# THE CANNABIS AND OLDER PERSONS STUDY: EXPANDING OUR MINDS THROUGH INTERDISCIPLINARY RESEARCH

**DOI:** 10.1093/geroni/igac059.392

**Published:** 2022-12-20

**Authors:** Brian Kaskie, Benjamin Han

## Abstract

The Cannabis and Older Persons Study has examined the increasing use of cannabis among Americans over 60 years old since 2016. This year’s symposium presents varied methodological approaches researchers have used to better understand the harms and benefits associated with cannabis use among older Americans. Divya Bhagianadh examines the association between cannabis legalization policies across the Untied States and corresponding use of end of life programs and services. Julie Bobitt analyzes interviews provided by older adults in Illinois and defines how attitudes held by medical doctors and other clinical care providers are critical to shaping cannabis use among older adults. Brian Kaskie considers the role of cannabis policy clusters. Jacobo Mintzer reviews findings from a controlled trial providing two cannabinoids to treat agitation among persons with Alzheimers in hospice. Thorsten Rudroff examines cannabis use among a sample of older persons at risk for falls. These studies reflect how cannabis use among older persons continues to grow and diversify, and how researchers have use different approaches to advance scientific understanding of determinants and outcomes, both desirable and undesirable, associated with cannabis use among older adults. This symposium offers policy makers and health care leaders a balanced perspective. On one side, we discuss how prevention and treatment efforts for substance use disorder, including cannabis use disorder, must increase proportionally to the increasing number of older cannabis users. On the other side, we consider how more than 5 million older Americans, especially those with pain, find some benefit in using cannabis.

